# Clinical performance of digital breast tomosynthesis–guided vacuum-assisted biopsy: a single-institution experience in Japan

**DOI:** 10.1186/s12880-022-00896-1

**Published:** 2023-01-05

**Authors:** Mirai Ido, Masayuki Saito, Hirona Banno, Yukie Ito, Manami Goto, Takahito Ando, Junko Kousaka, Yukako Mouri, Kimihito Fujii, Tsuneo Imai, Shogo Nakano, Kojiro Suzuki, Kenta Murotani

**Affiliations:** 1 Division of Breast and Endocrine Surgery, Department of Surgery, Aichi Medical University, 1-1 Yazakokarimata, Nagakute-City, Aichi 480-1195 Japan; 2 Department of Radiology, Aichi Medical University, 1-1 Yazakokarimata, Nagakute-City, Aichi 480-1195 Japan; 3 Biostatistic Center, Graduate School of Medicine, Kurume University, 67 Asahi-machi Kurume, Fukuoka, 80-0011 Japan

**Keywords:** Digital breast tomosynthesis, Breast cancer, Breast microcalcifications, Stereotactic biopsy, Digital breast tomosynthesis–guided vacuum-assisted biopsy

## Abstract

**Background:**

The purpose of this study was to evaluate the clinical performance of Digital Breast Tomosynthesis guided vacuum-assisted biopsy (DBT-VAB) for microcalcifications in the breast.

**Methods:**

Retrospective review of 131 mammography-guided VABs at our institution were performed. All of the targets were calcification lesion suspicious for cancer. 45 consecutive stereotactic vacuum-assisted biopsies (ST-VABs) and 86 consecutive DBT-VABs were compared. Written informed consent was obtained. Tissue sampling methods and materials were the same with both systems. Student’s t-test was used to compare procedure time and the Fisher’s exact test was used to compare success rate, complications, and histopathologic findings for the 2 methods.

**Results:**

The tissue sampling success rate was 95.6% for ST-VAB (43/45) and 97.7% (84/86) for DBT-VAB. Time for positioning (10.6 ± 6.4 vs. 6.7 ± 5.3 min), time for biopsy (33.4 ± 13.1 vs. 22.5 ± 13.1 min), and overall procedure time (66.6 ± 16.6 min vs. 54.5 ± 13.0 min) were substantially shorter with DBT-VAB (*P* < 0.0001). There were no differences in the distribution of pathological findings between the 2 groups.

**Conclusion:**

Depth information and stable visibility of the target provided by DBT images led to quick decisions about target coordinates and improved the clinical performance of microcalcification biopsies.

## Background

Digital breast tomosynthesis (DBT) provides a series of low-dose mammograms at various angles through the breast, reducing the effect of tissue overlap and improving visualization of malignancies [1–3]. Multiple studies have reported that DBT in conjunction with conventional mammography screening improves lesion characterization, which increases cancer detection and reduces false-positive results [4–11]. Some previous reports suggest that full-field digital mammography (FFDM) is more sensitive and specific than DBT for the detection of microcalcifications [12–14], but other reports provide conflicting data [15].

For mammographically detected lesions, including microcalcifications that cannot be identified on ultrasound, stereotactic vacuum-assisted biopsy (ST-VAB) is performed for histological diagnosis, which is the gold standard. DBT-guided vacuum-assisted biopsy (DBT-VAB), which has been developed over the past few years, can be performed in a similar manner as ST-VAB but it overcomes some of the limitations of ST-VAB [16–21].

The main difference between ST-VAB and DBT-VAB is the system used for targeting the lesion. Since one-view mammography does not provide information about depth along the z-axis, triangulation is required with ST-VAB to determine the depth of the target, commonly referred to as the Z coordinate. This process is sometimes cumbersome and time-consuming because the determined target looks different in a pair of stereotactic images (stereo pair shooting), resulting in more time needed to identify the target and occasional coordinate miscalculation. On the other hand, a tomoscout, which is an image from DBT, provides depth information without triangulation. Tomoscout is an imaging method in 3D mammography that produces several low-dose projections at various angles. Three-dimensional (3D) values (x, y, and z coordinates) are determined immediately by scrolling through the slice of each image and making a click where the target looks clearest [19, 20]. As more institutions implement DBT-VAB, it is important to understand the differences between the two systems. Some studies have investigated the clinical performance of DBT-VAB for calcified lesions and architectural disorders that are not detected on ultrasonography. These studies have shown the superiority of DVT-VAB over ST-VAB in terms of shorter examination time, simplicity, lower exposure dose, and fewer complications [16–22]. In most studies, biopsies are performed in the prone position for ST and in the sitting or lateral decubitus position for DBT [16, 17, 20]. However, no studies comparing DBT-VAB and ST-VAB in the same upright position have been reported. Furthermore, there have been no studies in Japanese women. The purpose of this study was to compare the clinical performance and effectiveness of DBT-VAB and ST-VAB in Japanese women.

## Materials & methods

### Mammography

**Fig. 1:**
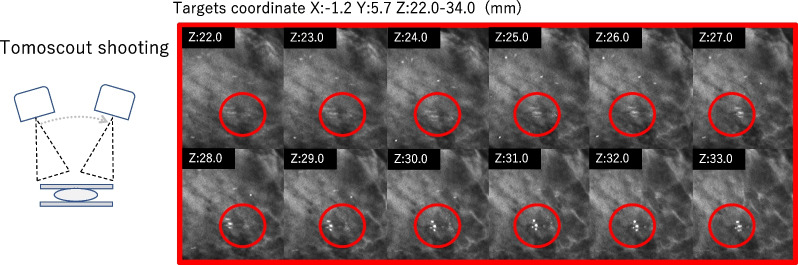
3D mammography images and tomoscout shooting of a calcified target. Thin-slice tomograms are reconstructed from one tomoscout shooting, in which 15 low-dose projections are taken with ± 7.5 degrees of turning. The target calcified lesion is identified by scrolling through the tomographic views

**Fig. 2:**
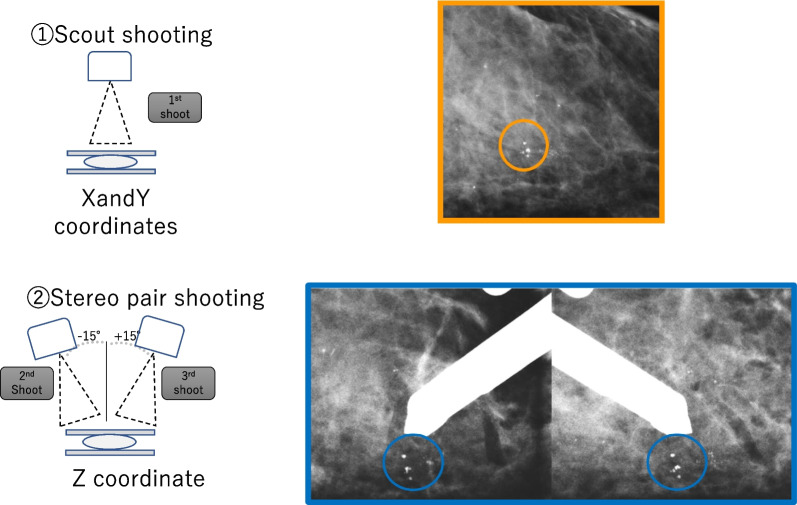
2D mammography images and targeting using stereo pair shooting. One-view mammogram providing a flat value (x and y coordinates) after stereo pair shooting. Two images of the target with ± 15 degrees of turning are used to calculate the depth of the target. (z coordinate) using triangulation

Selenia Dimensions (Selenia; Hologic, Bedford, MA, USA) was the mammography system used in this this study. In 3D mammography, thin-slice tomograms are reconstructed by taking 15 low-dose projections in 3.7 s with ± 7.5 degree turns [1, 2]. The reconstruction method was iterative super-resolution reconstruction (ISR). The target is identified by scrolling through these tomographic views (Fig. 1). By contrast, the conventional targeting technique requires 3 separate sets of two-dimensional (2D) mammography images. After scout shooting at 0 degrees for determining the flat location, i.e., the x and y coordinates, two stereo shooting turning angles at − 15 degrees and + 15 degrees are used to calculate the depth, or location of the z coordinate (Fig. 2) [20].

### Procedures and devices

**Fig. 3:**
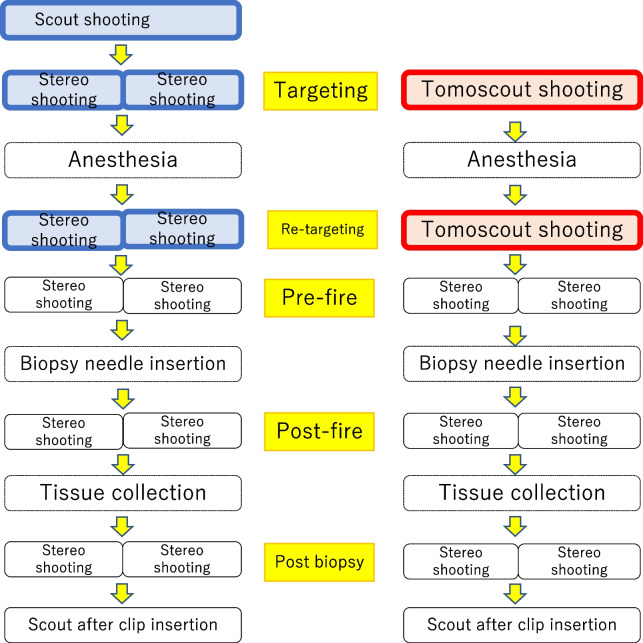
ST-VAB and DBT-VAB procedures

Both DBT-VAB and ST-VAB were performed in a seated position on a dedicated armchair. Each procedure is shown step-by-step in Fig. 3. After the breast is compressed and fixed using a dedicated fenestrated compression plate, the lesion is identified and placed in the center of the window. The target coordinates, determined by scrolling through the slices of each lesion and noting where the target looks the clearest, were again determined using the same procedure after skin disinfection and administration of local anesthesia for re-targeting. Injection of local anesthesia (total volume of 20 ml, consisting of 10 ml of 1% lidocaine with epinephrine and 10 ml of 1% lidocaine without epinephrine) along the expected needle track often displaces the target and changes its appearance. When the lesion appeared the same in multiple images, we chose the slice with the lesion depicting its characteristic shape to avoid losing sight of it after local anesthesia. We also took into account areas where more lesions are likely to be sampled. For breast biopsies, a guidance system (Affirm; Hologic) is installed as an add-on and Mammotome ST with an 11G Bladed Probe (Devicor Medical Products, Buffalo Grove, IL, USA) is used. Biopsies are performed through the shortest route to access the target lesions. Because metallic halation interrupts tomoscout shooting after biopsy needle insertion, we could not use stereo shooting for the rest of the procedure. Positioning is confirmed with pre-fire and post-fire 2D stereotactic image pairs. The Mammotome MicroMark clipping marker (Devicor Medical Products) is deployed after biopsy and confirmed with a final 2D image. A specimen radiograph is obtained to confirm the presence of calcification.

### Study population and target lesions

To compare the clinical performance of ST-VAB versus DBT-VAB, mammography-guided VAB was performed for 131 lesions. This preliminary retrospective study was approved by our hospital’s institutional review board of Aichi Medical University Hospital (approval number 2020-133). At the time of the procedure, standard written informed consent was obtained. We performed 45 ST-VAB procedures in 42 patients (45 lesions; median age, 50 years; age range, 36–70 years; 23 left breast lesions and 22 right breast lesions) from March 2013 to August 2015, the date of tomosynthesis implementation. From January 2016 to December 2018, we performed 86 DBT-VAB procedures in 86 patients (86 lesions, consisting of 39 left breast lesions and 47 right breast lesions). The median age of the patients was 49 years (range, 30–80 years). Patients included in this study were either recalled for breast cancer screening or referred by general practitioners. Most lesions were BI-RAD 4 or 5. Some lesions were worsening BI-RAD 3 or calcified lesions in patients with a history of breast cancer. Calcified lesions and breast density were classified according to the Breast Imaging Reporting and Data System (BI-RADS) established by the American College of Radiology as mammographic assessment categories. Breast density and microcalcification morphology were evaluated by 2 different surgeons who were engaged in screening mammography.

### Data collection and analysis

Both VAB systems used the same tissue sampling methods in the seated position. All biopsies were performed by 2 of 9 well-trained breast radiologic technologists and 1 breast surgeon. For each biopsy, tissue sampling success rate, technical problems, procedure time (time for positioning, time for targeting, and overall procedure time), complications (pain, infection, vasovagal reaction, large hematoma, and bleeding), and histopathologic findings were evaluated. Positioning time referred to the time from when the patient was seated in the chair to complete compression of the breast and verification that the lesion was visualized on the shooting range. Targeting time referred to the time for determining the target x, y, and z coordinates using 2D or 3D mammography after biopsy and inserting the clip into the breast. Overall biopsy time referred to the time from when the patient entered the examination room to when the patient left the room. Pathological diagnosis was based on the 2012 World Health Organization Classification of Tumors of the Breast [25]. For simplification, we classified lesions into four categories: benign, high risk, malignant in situ, and malignant invasive. Final surgical pathology results were compared with biopsy results to evaluate the upgrade rate.

### Statistical analysis

Student’s t-test was used to compare patient demographics and procedure time. Fisher’s exact test was used to compare success rate, distribution of lesion types, and pathological findings. *P* < 0.05 was considered to indicate a statistically significant difference. For both systems, 95% confidence intervals were calculated. All statistical analyses were performed using EZR (Saitama Medical Center, Jichi Medical University, Saitama, Japan), which is a graphical user interface for R (The R Foundation for Statistical Computing, Vienna, Austria), and SAS 9.4 (SAS Institute, Cary, NC, USA).

## Results

### Patient and lesion characteristics

Clinical performance was better with DBT-VAB compared with ST-VAB in terms of procedure time and complication rate. There were no differences in patient age (*P* = 0.759), breast density (*P* = 0.975), past or simultaneous history of breast cancer (*P* = 0.758), calcified lesion morphology (*P* = 0.316), or calcified lesion distribution (*P* = 0.681) between the 2 groups. Background demographic characteristics of the patients are shown in Tables 1 and 2.

**Table 1:** Background of patients with calcified lesions who underwent ST-VAB or DBT-VAB

	ST-VAB (n = 45)	DBT-VAB (n = 86)	*P* Value
Mean age*	50.1 ± 9.3	50.7 ± 10.3	0.76
*Breast density*			0.98
Almost entirely fatty	1	2	
Scattered areas of fibroglanudular density	3	5	
Heterogeneously dense	34	63	
Extremely dense	7	16	
*History of breast cancer*			0.13
No history	38	80	
Prior or simultaneous history	7	6	

Data are numbers of patients. *Data are means ± standard deviations

ST-VAB = stereotactic vacuum-assisted breast biopsy, DBT-VAB = digital breast tomosynthesis vacuum-assisted breast biopsy

**Table 2:** Characteristics of calcified lesions biopsied with ST-VAB versus DBT-VAB

	ST-VAB (n = 45)	DBT-VAB (n = 86)	*P* Value
*Distribution*			0.72
Diffuse	0	1	
Regional	6	7	
Grouped	32	66	
Linear	0	0	
Segmental	7	12	
*Morphology*			0.37
Round	0	5	
Coarse Heterogeneous	13	15	
Amorphous	13	27	
Fine Pleomorphic	16	31	
Fine linear or Fine-linear branching	3	8	

### Technical success rate

The tissue sampling rate was 95.6% (43/45) for ST-VAB and 96.5% (83/86) for DBT-VAB (*P* = 1.00). Two ST-VABs were aborted, because of vasovagal reaction and patient inability to tolerate the procedure in 1 case and skin penetration during biopsy in the other. One DBT-VAB was aborted because the target became obscured after injecting local anesthesia. There were two sampling errors with DBT-VAB, which were diagnosed as “no malignancy” with a comment of no calcification in the specimen or insufficient material for histological diagnosis (Table 3). Surgical excision or careful mammographic follow-up was performed in cases of aborted biopsy or sampling error. Other technical problems during the procedure included re-positioning, re-insertion of the biopsy needle, and broken marking clip upon replacement in DBT-VAB, but all biopsies were completed.

**Table 3:** Results of tissue sampling rate for ST-VAB versus DBT-VAB

	ST-VAB (n = 45)	DBT-VAB (n = 86)	*P* value
Success	43(95.6)	83(96.5)	1.00
Technical trouble	1	1	1.00
Patient tolerance	1	0	0.34
Sampling error	0	2	0.55

### Time needed to perform biopsy

Time for positioning, time for targeting, and overall procedure time were compared between the 2 systems. With ST-VAB, these times were 10.6 ± 6.4 min, 33.4 ± 13.1 min, and 66.6 ± 16.6 min, respectively. With DBT-VAB, these times were 6.7 ± 5.3 min, 22.5 ± 13.1 min, and 54.5 ± 13.0 min, respectively. Each type of procedure time was shorter with DBT-VAB (*P* < 0.0001) (Table 4).

**Table 4:** Procedure time for ST-VAB versus DBT-VAB

Time for procedure	ST-VAB (n = 45)	DBT-VAB (n = 86)	*P* Value
Positioning*	10.6 ± 6.4	6.7 ± 5.3	< 0.0001
Targeting*	33.4 ± 13.1	22.5 ± 13.1	< 0.0001
Entire biopsy*	66.6 ± 16.6	54.5 ± 13.0	< 0.0001

^*^Data are means ± standard deviations

### Patient tolerance and complications

No major complications were observed with either system. Six patients who underwent ST-VAB and 2 patients who underwent DBT-VAB developed vasovagal reactions (*P* = 0.0196). Most vasovagal reactions were self-limited but a patient in the ST-VAB group was unable to continue, as mentioned above. Three patients who underwent DBT-VAB complained of severe pain during the biopsy and required additional local anesthesia for pain control or had fewer specimens taken than usual. No infections requiring antibiotics or bleeding requiring intervention was observed with either system (Table 5).

**Table 5:** Comparison of complications with ST-VAB versus DBT-VAB

	ST-VAB (n = 45)	DBT-VAB (n = 86)	*P* Value
Vasovagal reaction	6(1 aborted)	2	0.0196
Pain	0	3	0.551
Infection	0	0	–
Bleeding/hematoma	0	0	–

### Histopathology

No differences were found in the distribution of pathological findings by VAB system (*P* = 0.452). Nearly one-third of biopsies yielded malignant results in both groups: 15 of 43 ST-VABs and 23 of 85 DBT-VABs (*P* = 0.843). Subsequent surgical excision was performed in 14 patients who underwent ST-VAB and 25 patients who underwent DBT-VAB. The histological upgrade rate was 4.4% (2/43) for ST-VAB and 8.2% (7/85) for DBT-VAB (*P* = 0.717) (Table 6). Surgical resection was performed in 15 cases of ST-VAB and 23 cases of DBT-VAB. Pathological findings of surgically resected lesions in cases diagnosed with ST-VAB were invasive ductal carcinoma (IDC) (n = 3), low-grade ductal carcinoma in situ (DCIS) (n = 2), intermediate DCIS (n = 7), and high-grade DCIS (n = 3). Average tumor size was 13.1 mm (range, 5–25 mm). Hormone receptors were positive in all lesions except for two lesions with missing information on hormone receptor status. HER2 status was negative in all three IDC cases. Pathological findings of surgically resected lesions in cases diagnosed by DVT-VAB were IDC (n = 6), low-grade DCIS (n = 3), intermediate DCIS (n = 5), high-grade DCIS (n = 4), atypical ductal hyperplasia (ADH) (n = 1), and lobular carcinoma in situ (LCIS) (n = 1). In one case, there were no lesions remaining within the surgically resected specimen. Two patients underwent surgery at other hospitals and pathological findings were not available. Based on the information available, average tumor size was 25.6 mm (range, 0.7–85 mm). Hormone receptors were positive in 14 cases and negative in 1 case. Of the six IDC cases, four were HER2 positive and two were HER2 negative. The pathological criteria were based on the previous report [26].

**Table 6:** Histopathologic results for ST-VAB versus DBT-VAB

	ST-VAB (n = 43)	DBT-VAB (n = 85)	*P* Value
*Histopathology*			0.452
*Benign*	23	56	
*High risk*	8	6	
*Malignant*			
In Situ	11	20	
Invasive		3	
Surgical resection	15	23	0.843
Upgrading	2	7	0.717

## Discussion

We confirmed the superiority of DBT-VAB over ST-VAB in this study. The main difference was the method used to determine target coordinates. Triangulation, which was a time-consuming process for targeting, was omitted in DBT-VAB in this study. The operator may fail to identify the same lesion in a pair of stereotactic images; this failure results in a miscalculation of lesion depth [19]. The improved visualization of microcalcifications in DBT images might be one reason for improvements in clinical biopsy characteristics, especially procedure time. In a previous study, DBT-VAB was associated with higher technical success rates, shorter procedure times, and less radiation exposures, which was similar to our results [16].

The detection and evaluation of clustered microcalcifications is an important component of mammographic analysis. However, there is some concern that DBT might not depict microcalcifications to the same extent as FFDM [14]. Some authors have reported that clustered or faint microcalcifications can be overlooked in DBT because they are spread in different slices and are seen with greater clarity or higher sensitivity on conventional mammography. These microcalcifications might occasionally be missed or understaged with DBT-only screening. [12, 13, 15]. Variations in the conspicuity of microcalcifications might result from computational reconstruction [22]. Moreover, spatial resolution is lower with DBT owing to tube motion, greater pixel size, and pixel binning, which also affect lesion conspicuity [14]. Exposure to a low radiation dose in each slice and longer time for tomoscout acquisition may also contribute to this result. Since a tomoscout takes a longer exposure time per acquisition (approximately 7 s), motion artifact due to slight patient movements sometimes causes target blurriness. This was the reason for sampling error in our study. Although we managed to target lesions that were barely visible and continued the procedure, we were unable to obtain the target lesions in the specimen. Integrating DBT with FFDM is required to compensate for this problem, but computer-aided detection (CAD) or synthesized mammography, which is a technology for synthesizing 2D images generated from DBT data, might compensate for this limitation.

However, once the target has been detected, visibility was relatively stable and clear in the tomoscout images. The target figuration sometimes looks different from the stereo pair shooting image because of differences in shooting angle. This improvement in visibility might lead to fewer miscalculations of the target coordinates and shorter procedure times, resulting in fewer biopsy complications. In our experience with DBT-VAB, very fine targets occasionally become blurry and difficult to visualize after anesthesia administration or with inadequate breast fixation. Fine targets that are hard to identify in the tomoscout were not able to be identified even after shifting to conventional 2D stereo pair shooting.

Some previous studies have found that lesion targeting requires significantly less time with DBT-VAB and has better clinical performance than conventional ST-VAB [16, 19, 20]. In our study, DBT-VAB was associated with significantly shorter biopsy time. Although there were no differences in the positioning method for the two systems, obtaining a tomoscout image in advance of biopsy provides information on breast thickness and precise information on lesion depth. This information helped us predict the risk of skin perforation, calculate safety margins, and shorten the time for setting the target in the middle of the penetration window. Consequently, it shortened positioning time. In addition, omitting the triangulation procedure for determining the target coordinates decreases breast compression time and overall procedure time, resulting in reduced physical burden on both patients and medical staff.

No bleeding or hematomas were observed after biopsy with either system. We believe this is due to appropriate quantities of anesthesia with epinephrine and adequate astriction after biopsy. Pain after needle insertion is a complication we surely want to avoid, because additional anesthesia after needle insertion might change the distance to the target, which may require re-insertion of the needle. We should be careful about injecting anesthesia into an appropriate area around the target, with consideration of target movement due to anesthesia. Although vasovagal reactions were self-limited in most cases, they do occur at a certain rate. Scharding et al. found that shorter procedure time with DBT-VAB may contribute to fewer vasovagal reactions and higher patient tolerance of the procedure [20]. In addition, shorter biopsy time improves patient compliance and results in fewer movement artifacts [19]**.** However, considering the low numbers of vasovagal reactions in the 2 groups, it is difficult to state whether the difference was due to chance or represents an actual difference.

Radiation exposure during biopsy was not recorded in our study, but Bahl et al. and Viala et al. reported less exposure with DBT-VAB [16, 21]. The actual radiation dose with mammography varies substantially depending on breast size and glandular and adipose composition [9]. For the breast phantom representing the average breast with a compressed thickness of 5 cm and a 50% glandular fraction, the mean glandular dose (MGD) of FFDM and DBT was 1.2 mGy and 1.3 mGy per view, respectively [23]. ST-VAB requires at least 11 sets of 2D scouts, whereas DBT-VAB requires at least 6 sets of 2D scouts and two sets of 3D tomoscouts. If we calculate the radiation dose of these VAB systems with this information, the radiation dose is 13.2 mGy for ST-VAB and 10.4 mGy for DBT-VAB. Thus, DBT-VAB might lower the patient’s radiation dose during biopsy. As a side note, adding DBT to FFDM more than doubles the radiation dose needed for breast screening. However, recent studies have shown that the screening performance of reconstructed synthetic 2D images plus DBT is not inferior to the performance with FFDM plus DBT [24]. Synthetic 2D images may replace FFDM with a remarkable dose reduction for screening.

Our study had several limitations. The small number of patients who underwent biopsy limits the generalizability of our conclusions. The results of our retrospective comparison of ST-VAB and DBT-VAB may have biases because biopsy system was not randomized, and different systems were used during different time periods. Operator experience is another limitation. Less experienced operators tend to misidentify the target because the determined target looks different in a pair of stereotactic images; this failure results in a miscalculation of lesion depth and might underestimate the clinical performance of ST-VAB. Not all lesions underwent surgical resection and histological examination. The possibility of upgrade for non-resected lesions is unclear. Although the difference between the two systems with regard to histological upgrade was insignificant, it will be a major limitation when more biopsies are being performed. Further studies are needed to evaluate clinical outcomes.

## Conclusion

DBT imaging improves the visibility of the calcifications compared to conventional stereo pair shooting imaging. This improvement in visibility and depth information in advance resulted in quick and accurate targeting, thereby requiring significantly shorter biopsy planning time and overall procedure time. We believe that DBT-VAB had superior clinical performance compared with ST-VAB and may reduce the stress of both medical staff and patients.

## Data Availability

The datasets used and analysed during the current study are available from the corresponding author on reasonable request.

## Abbreviations

DBT: Digital breast tomosynthesis
FFDM: Full-field digital mammography
ST-VAB: Stereotactic vacuum-assisted biopsy
DBT-VAB: DBT-guided vacuum-assisted biopsy
IDC: Invasive ductal carcinoma
DCIS: Ductal carcinoma in situ
